# Functional popliteal angle tests improve identification of short hamstring muscle–tendon length in patients with a central neurological lesion

**DOI:** 10.1038/s41598-023-47667-8

**Published:** 2023-11-22

**Authors:** Mahdieh Hajibozorgi, Ilse Leijen, Juha M. Hijmans, Christian Greve

**Affiliations:** 1 Department of Rehabilitation Medicine, University of Groningen, University Medical Center Groningen, Groningen, The Netherlands; 2 Center for Human Movement Sciences, University of Groningen, University Medical Center Groningen, Groningen, The Netherlands

**Keywords:** Rehabilitation, Preclinical research, Orthopaedics

## Abstract

This study introduces a functional exercise protocol to improve the identification for short hamstring muscle–tendon length (HMTL), a common contributor to crouch gait in patients with central neurological lesions (CNL). The functional exercise protocol incorporates a knee extension movement with hip in a flexed position, while standing on one leg (functional popliteal angle test) and walking with large steps to the current standard protocol (walking at comfortable speed and as fast as possible). The main aim was to establish whether the new protocol allows better determination of maximum HMTLs and diagnostics of short HMTL in patients with a CNL. Lower limb 3D marker position data from 39 patient limbs and 10 healthy limbs performing the exercises were processed in OpenSim to extract HMTLs. The new protocol provoked significantly larger HMTLs compared to the current standard protocol. The total number of limbs classified as having too short HMTLs reduced from 16 to 4 out of a total of 30 limbs walking in crouch. The new protocol improves determination of maximum HMTL, thereby improving short HMTL diagnostics and identification of patients in need of lengthening treatment. Inter-individual variability observed among patients, indicating the need to include all exercises for comprehensive diagnosis.

## Introduction

Crouch gait is a prevalent abnormality in individuals with central neurological lesions (CNL). About 75% of all patients with cerebral palsy (CP) walk with increased hip and knee flexion at initial contact and during the stance phase of gait^1–6^. This inefficient walking pattern increases hip and knee joint loads compared to a normal, healthy walking pattern^7^. Patients walking in crouch are slower, cover shorter distances and have an increased risk of joint discomfort and early joint degeneration^8,9^. Without intervention, crouch gait might deteriorate over time and lead to a loss of independent walking ability^3,10,11^. To treat crouch gait and facilitate a more normal, healthier walking pattern is therefore one of the main aims in clinical gait rehabilitation of patients with a CNL.

Effectively treating crouch gait depends on early and accurate diagnosis of its underlying cause. However, identifying the underlying cause is challenging since it is usually multi-factorial and can involve any combination of deficits in hamstring muscle–tendon length (HMTL) or spasticity, increased anterior pelvic tilt (e.g., due to short or spastic hip flexor muscles), ankle plantar flexor and hip extensor muscle weakness, or impaired hip and knee joint mobility^6^. One of the most frequently diagnosed causes of crouch gait is short HMTL^6^, which impairs knee-extension and hip-flexion in terminal swing, leading to increased knee-flexion at initial contact.

Currently, short HMTL is diagnosed using a combination of instrumented gait assessments (3D clinical gait analysis), musculoskeletal modeling, and a physical examination of hip and knee joint mobility^12–14^. By comparing the patient's HMTL at initial contact with normative data from healthy control subjects, patients can be classified as having short HMTL and requiring lengthening treatment. However, current treatment outcomes after hamstring lengthening are unsatisfactory in many patients^13,15–19^. For instance, across scientific studies gait improvements post-hamstring muscle lengthening surgery were observed in 50% to 70% of patients, yet it deteriorated in about 15%^13,15,17^. A possible reason for the unsatisfactory results after lengthening treatment might be diagnostic inaccuracies. We propose that the current 3D clinical gait analysis protocol executed with patients walking at comfortable speeds does not provoking maximum HMTL resulting in false positive diagnoses of short HMTLs^20,21^.

To improve the diagnostic accuracy of short HMTL, fast walking conditions have been incorporated recently into 3D clinical gait analysis protocols^22^. The underlying premise is that patients increase gait speed by increasing step length through more hip-flexion and less knee-flexion of the leading limb at initial contact^22,23^. More hip-flexion and less knee-flexion at initial contact would in turn provoke larger HMTL compared to walking at comfortable speeds and allow better determination of a patient’s maximum HMTL^21–24^. Better determination of maximum HMTL in turn would improve diagnostic accuracy of short HMTL and lead to fewer false positive diagnoses. However, patients with a CNL often suffer from multiple neuromuscular impairments possibly impairing their ability to increase gait speed by taking larger steps. For example spasticity, limitations in contralateral hip-extension joint range of motion, ipsilateral weakness of the hip and calf muscles, or balance deficits might impair a patient to increase gait speed through taking large steps and utilize maximum HMTL during 3D clinical gait analysis.

We address the limitations in current diagnostics of short HMTL by introducing a new exercise protocol for short HMTL diagnostics. This protocol adds a functional popliteal angle test (a combined hip-flexion and knee-extension motion while standing supported by a caregiver on one leg) performed slowly and as fast as possible and walking with large steps to the current standard protocol. For each test, HMTLs were computed from 3D marker position data and OpenSim musculoskeletal modeling software^25^. The main aim of this study was to establish whether the new functional exercise protocol is a better determinant of maximum HMTL as compared to walking at comfortable and fast speeds, and can improve diagnostic accuracy of short HMTL in children and adults with a CNL. Based on biomechanics and anatomy, we propose two hypotheses: (1) the functional popliteal angle test and walking with large steps will result in larger maximum HMTL values than walking at a comfortable speed and walking fast, (2) our new diagnostic protocol will lead to fewer patients being categorized as having short HMTL compared to the number of positive diagnoses when considering walking at comfortable and fast speeds only. In addition, normative values for each of the exercises from healthy young adults will be established.

## Methods

### Study design

To validate the new diagnostic exercise protocol, we performed a combined retrospective analysis of patients’ recordings and prospective experimental study on healthy young adults. Both study parts were approved by the medical ethics review committee of the University of Medical Center Groningen (number 2022.228 and 2022.243). All measurements were performed at the Motion Laboratory of the University Medical Center Groningen, Department of Rehabilitation Medicine, in accordance with the guidelines approved by the ethical committee. The current study is part of a larger project and only those details relevant to this paper are mentioned. For the entire experimental protocol please see the Supplementary Information.

### The new functional exercise protocol

The new functional exercise protocol consists of five exercises including walking at comfortable speed, walking as fast as possible, walking with large steps, and performing a functional popliteal angle test slowly and as fast as possible. The functional popliteal angle test, an active adaptation of the traditional passive popliteal angle test, requires participants to stand on one leg, flex the hip of the other leg to about 90°, and then actively attempt maximal knee extension while keeping the hip flexed. Each walking condition is to be performed four times, yielding 15–20 steps per condition. Between each walking condition there is a rest period of 1–2 min. During all the exercises the patient is barefoot. During the functional popliteal angle test, the patient is given assistance to counteract any balance deficits that might affect their ability to perform the test. The final step is a physical assessment including the (original, unilateral) passive popliteal angle test by a qualified physical examiner. A detailed description of the entire exercise protocol can be found in the Supplementary Information.

### Retrospective patient inclusion

Patients with a CNL who signed informed consent during a regular visit for clinical 3D gait analysis at the motion laboratory of the Department of Rehabilitation Medicine, UMCG Groningen were screened for eligibility. If a patient was younger than 12 years old, their parents had provided the informed consent. Patients were eligible for inclusion if (a) they were diagnosed with a CNL and were referred to the motion laboratory for assessments of crouch gait or deficits in HMTL by their treating rehabilitation physician, (b) received 3D clinical gait analysis including the functional popliteal angle test, walking at comfortable speed and as fast as possible and/or walking with large steps. Patients who were unable to perform any of the new exercises in the protocol (walking with large steps and the slow and fast functional popliteal angle tests) were excluded from the analysis. For children with a unilateral CNL, only the affected side was evaluated. In cases of bilateral CNL, both sides were included in the final data analysis as separate limbs.

### Prospective study in healthy participants

Ten healthy participants were recruited to acquire normative values of HMTL. The inclusion criteria were: aged 18 to 65 years, no self-reported musculoskeletal impairments impacting walking performance. No additional exclusion criteria were used. Eligible participants signed written informed consent. To make a meaningful comparison between healthy participants and patients, we aimed to identify a gait speed that closely matched the patients' comfortable walking speed when normalized to femur length. Therefore, the healthy participants performed the same exercises as the patients, except that walking at comfortable speed was replaced by walking at five fixed speeds (0.7 m/s, 0.9 m/s, 1.1 m/s, 1.3 m/s, and 1.5 m/s representing 55%, 70%, 85%, 100%, 115% of healthy individuals' standard comfortable walking speed^26^). Before the experiments, body height, body mass, ankle width, knee width, and leg length were measured to run the plug-in-gait modelling pipelines.

### Data acquisition

3D marker position data were acquired with 10 Vicon cameras (Vero, Oxford, United Kingdom) and the Nexus 2.14.0 software. Ground reaction force data were acquired using two AMTI force plates (Advanced Mechanical Technology, Inc., Watertown, USA). The retrospective data were acquired during usual clinical care. The marker setup consisted of 18 reflective markers, including 16 markers from the Plug-in-Gait Lower Body model. During the static calibration, two additional markers were placed, one on the medial side of each knee. These markers, along with the femur markers, were used to align the knee flexion–extension axis and were removed after calibration.

The same workflow to calibrate the knee axis was used for the healthy participants. A custom-made MATLAB script was used to control walking speed during prospective data acquisition, allowing a 10% deviation from the intended gait speed. The order of walking conditions for each healthy participant was randomized using a custom MATLAB script. Functional popliteal angle tests were always performed after walking conditions and with the participant’s dominant leg.

### Data analysis

Custom-made Python (3.0) scripts were used to run standard OpenSim (4.3) modelling workflows of scaling, inverse kinematics, and muscle analysis to estimate maximum HMTLs. These workflows were performed on the original gait-2392 model. The semitendinosus, semimembranosus, and biceps femoris long head were analyzed for HMTLs. Given that previous research has indicated similar length changes between the semimembranosus and semitendinosus muscles during walking^27^, as well as our own analyses across five exercises showing similar length changes between the medial and lateral hamstring muscles, semitendinosus data were used for the remainder of the manuscript. Further details of length changes of different hamstring muscles are available in the Supplementary Information. The HMTL retrieved from the OpenSim muscle analysis tool was normalized by each participant’s individual femur length^28^. The femur length was determined as the distance between the right/left hip joint centers (RFEP/LFEP virtual marker) and the right/left knee joint center (RFEO/LFEO virtual marker) from the Plug-in-Gait model.

For each participant, the maximum hamstring muscle–tendon length (HMTL_max_) was determined as follows: For slow and fast popliteal angle tests, the maximum HMTL was calculated across 3 to 5 repetitions. For each experimental walking condition, the maximum of peak HMTL was calculated across gait cycles within a gait trial and gait trials within one walking condition (e.g., walking with large steps). Subsequently, HMTL excursion (HMTL_exc_) for each walking condition was determined by subtracting the average of the minimum HMTL from the average of the maximum HMTL across gait cycles within a gait trial and gait trials within one walking condition. Additionally, HMTL reserve capacity (HMTL_rc_) was calculated as follows:

1 $${HMTL}_{rc}=(\frac{{HMTL}_{max}^{*}-{HMTL}_{max}^{w}}{{HMTL}_{max}^{*}})\times 100$$

where ${\mathrm{HMTL}}_{\mathrm{max}}^{*}$ is the largest HMTL_max_ observed across all exercises and ${\mathrm{HMTL}}_{\mathrm{max}}^{\mathrm{w}}$ is the HMTL_max_ when walking at a comfortable speed (patients) or 1.3 m/s (healthy participants).

Next to muscle–tendon parameters, gait speed, step length, cadence, as well as hip and knee joint angles, and pelvis tilt angles at initial contact were analyzed to describe the patients’ and healthy participants’ gait pattern. Gait speed was determined by measuring the distance traveled by the virtual PELO (pelvic origin) marker from the Plug-in-Gait model during one trial and dividing it by the trial duration. Gait speed and step length were normalized to the leg length. Only data from the dominant limb of healthy participants and the affected limb in patients with unilateral CNL were used for further analysis. In case of bilateral CNL, both limbs were chosen for further analysis. Trials with non-physiological HMTL changes or joint kinematics due to missing data points exceeding 10 consecutive frames were excluded from the final analysis.

To better account for between-subject differences we computed patient specific normative HMTL_max_ values by imposing healthy lower limb kinematics from a single gait cycle of a representative healthy participant to the patients’ scaled OpenSim model (see the Supplementary Information for joint kinematics of the representative participant and the Inverse Kinematics set-up file). The newly generated normative muscle–tendon length data is referred to as “normative HMTL” in the remaining manuscript. The patient-specific normative HMTL_max_ data was validated through comparison with normative values from the healthy participants (Supplementary Table 1).

### Sub-group analysis

Prior to statistical testing, sub-groups were formed based on the amount of knee flexion and anterior pelvis tilt at initial contact. “Crouch gait” and “increased anterior pelvis tilt” were defined retrospectively as a knee flexion angle and an anterior pelvis tilt angle at initial contact larger than the average knee flexion and anterior pelvis tilt angles from the healthy participants’ dataset plus two standard deviations. Specifically, crouch gait was identified when the knee flexion angle at initial contact exceeded 17.08°, and increased anterior pelvic tilt was identified when the angle exceeded 6.85°. Using these criteria, patient limbs were categorized into the following sub-groups: (1) Patient limbs exhibiting a gait pattern with crouch and without increased anterior pelvis tilt (CR) and (2) Patient limbs exhibiting a gait pattern with crouch and increased anterior pelvis tilt (CR-AT). It should be noted that patient limbs which exhibited a gait pattern with an increased anterior pelvis tilt but without crouch, or those which exhibited neither crouch nor increased anterior pelvis tilt, are not categorized into any of the defined sub-groups. For patients with bilateral CNL, it was possible that the two limbs from the same individual be categorized into different sub-groups.

### Statistics

A repeated measure ANOVA was performed with the HMTL_max_ as the dependent variable. Different exercises (walking at a comfortable speed, walking fast, walking with large steps, fast popliteal test, and slow popliteal test) served as the within-subjects factor, and participant type (healthy or patient) served as the between-subject factor. In our study, the patients' comfortable normalized walking speed was found to be 1.37 1/s (Table 2) and we found that the healthy participants’ gait speed of 1.1 m/s resulted in an average normalized speed of 1.33 1/s, closely matching the patients' comfortable speed. This statistical analysis aimed to investigate the main effect of participant type on the HMTL_max_ as well as the interaction effect between the participant type and exercise on HMTL_max_. The significance level was set at α = 0.05.

Due to the identification of a significant interaction effect between exercise and participant type (p < 0.001, α = 0.05), post-hoc comparisons with pairwise t-tests were conducted between the healthy and patient groups in each individual exercise. A Bonferroni correction was applied to control for Type I errors arising from multiple comparisons, resulting in α = 0.05/5 = 0.01 for each of the five comparisons. Additionally, within each participant group, t-tests were conducted between each pair of exercises to assess the significance of any observed differences in HMTL_max_. For these within-group comparisons, a Bonferroni correction was similarly applied, resulting in α = 0.05/10 = 0.005 for each of the ten comparisons.

To compare the HMTL_rc_ and ${\mathrm{HMTL}}_{\mathrm{max}}^{*}$ between the patient and healthy groups two separate independent-samples t-tests were conducted. The significance level for each test was set at α = 0.05. All statistical analyses were conducted using SPSS 29 (IBM Corporation Software Group, Somers, NY, USA).

## Results

Data from 22 individual patients were included in the final analysis. Three patients of these 22 were evaluated both before and after an intervention, resulting in a total of 25 datasets and 39 limbs. Because the interventions were targeted to increase HMTL, we consider them in this paper as separate datasets. One patient, accounting for 2 limbs, was unable to complete the slow and fast popliteal angle test. Of the 39 included patient limbs, 24 were categorized into the CR group and six into the CR-AT group. The remaining limbs did not fit into these subgroups; three limbs were categorized as exhibiting a gait pattern without crouch but with increased pelvic tilt and six were categorized as exhibiting a gait pattern with neither crouch nor increased anterior pelvic tilt. Table 1 presents the participant characteristics of all sub-groups as well as the passive popliteal angle test results. Among the 39 included patients' limbs, 31 belonged to patients with CP, four to patients with hereditary spastic paraplegia, two to a patient with spinal cord injuries, one to a patient with spastic paresis, and one to a patient with cerebral infarction.

**Table 1:** Descriptive of the included participants.

	Healthy	Patients	CR	CR-AT
Number of limbs	10	39	24	6
Gender (M/F)	4/6	23/16	13/11	3/3
Age (years) (SD)	23.9 (1.2)	16.4 (11.7)	17.2 (12.7)	17.8 (16.3)
Height (cm) (SD)	175.6 (9.2)	159.6 (19.1)	160.3 (19.4)	154.7 (13.5)
Weight (kg) (SD)	68.7 (10.0)	55.5 (21.6)	57.7 (24.3)	51.9 (21.9)
Passive popliteal angle (°) (SD)	40 (23)	64 (19)	64 (22)	70 (10)

### HMTL; group-level assessment

**Figure 1:**
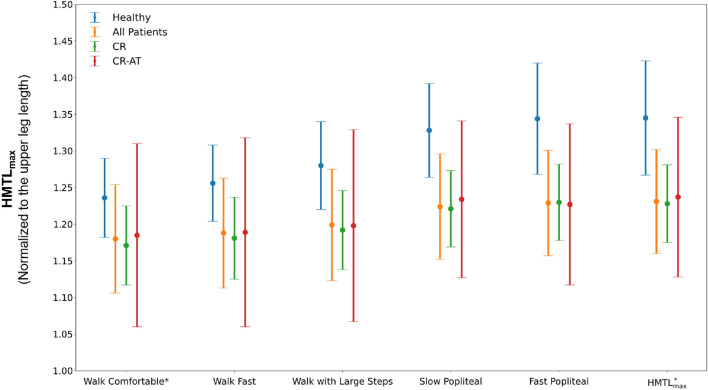
Average and standard deviation of HMTL_max_ normalized to the upper leg for healthy participants, all patients, CR, CR-AT, and AT group. For the healthy group, the "Walk Comfortable" data represents HMTL when walking speed is 1.1 m/s. This speed closely aligns with the average comfortable walking speed observed in the patient group. ${\mathrm{HMTL}}_{\mathrm{max}}^{*}$ indicates the maximum HMTL_max_ across all the exercises.

Across both the healthy and patient groups, HMTL_max_ increased with increasing gait speed. Furthermore, for healthy participants and patients, the HMTL_max_ was larger when walking with large steps (1.280 [SD = 0.060]/1.199 [SD = 0.076]) as compared to either walking at a comfortable speed for patients or 1.1 m/s for healthy participants (1.236 [SD = 0.054]/1.180 [SD = 0.074]), or walking fast (1.256 [SD = 0.052]/1.188 [SD = 0.075]). The largest HMTL_max_ were observed during the fast popliteal angle test, which were 1.229 [SD = 0.072] in patients and 1.344 [SD = 0.076] in healthy participants (Tables 2 and 3). Similar trends were observed within each sub-group except for CR-AT, where the largest HMTL_max_ was observed during the slow popliteal angle test and not the fast popliteal angle test (Fig. 1). Within the patient group, all exercise pairings showed significant differences in HMTL_max_ (p < 0.001, α = 0.005), except between the slow and fast popliteal angle test (p = 0.063, α = 0.005). Conversely, within the healthy group, all exercise pairings revealed significant differences (p < 0.001, α = 0.005), including between the slow and fast popliteal angle tests (p = 0.003, α = 0.005).

**Table 2:** Results of the patient group.

Exercise	Group subgroup	Gait speed (1/s) (SD)	Step length (–) (SD)	Cadence (1/s) (SD)	Knee flexion^1^ (°) (SD)	Hip flexion^1^ (°) (SD)	Pelvis tilt^1^ (°) (SD)	HMTL_max_ (–) (SD)	HMTL_excr_ (–) (SD)	${\mathrm{HMTL}}_{\mathrm{max}}^{*}$ is provoked^2^	Sufficient HMTL is exhibited^3^
Slow Popliteal	All patients				30.5 (13.6)	57.7 (11.2)		1.224 (0.072)		12/37	27/37
	CR				34.3 (14.6)	59.2 (11.2)		1.221 (0.052)		6/22	16/22
	CR-AT				25.6 (11.9)	55.0 (10.2)		1.234 (0.107)		4/6	5/6
Fast Popliteal	All patients				28.1 (12.9)	57.8 (12.0)		1.229 (0.072)		20/37	28/37
	CR				31.0 (13.5)	60.5 (11.5)		1.230 (0.052)		15/22	18/22
	CR-AT				29.2 (8.4)	56.1 (9.7)		1.227 (0.110)		0/6	5/6
Walk Comfortable	All patients	1.37 (0.30)	0.720 (0.112)	1.90 (0.22)	22.2 (8.3)	31.7 (7.6)	4.1 (4.3)	1.180 (0.074)	0.141 (0.031)	0/39	14/39
	CR	1.37 (0.33)	0.707 (0.116)	1.92 (0.24)	25.0 (7.8)	32.1 (6.3)	2.7 (2.8)	1.171 (0.054)	0.136 (0.024)	0/24	6/24
	CR-AT	1.35 (0.25)	0.705 (0.092)	1.91 (0.16)	24.8 (5.2)	34.7 (12.2)	9.4 (1.5)	1.185 (0.125)	0.154 (0.038)	0/6	3/6
Walk Fast	All patients	2.03(0.51)	0.841 (0.127)	2.43 (0.42)	23.4 (8.2)	34.3 (7.0)	4.9 (4.0)	1.188 (0.075)	0.153 (0.031)	1/39	18/39
	CR	2.02 (0.56)	0.828 (0.126)	2.45 (0.47)	26.5 (7.9)	35.4 (5.5)	3.6 (2.7)	1.181 (0.056)	0.150 (0.026)	1/24	10/24
	CR-AT	1.94 (0.44)	0.796 (0.094)	2.44 (0.43)	24.8 (4.0)	33.7 (11.4)	8.9 (3.1)	1.189 (0.129)	0.157 (0.041)	0/6	3/6
Walk with Large Steps	All patients	1.41 (0.33)	0.910 (0.225)	1.57 (0.22)	23.3 (8.9)	35.7 (12.8)	3.4 (6.0)	1.199 (0.076)	0.155 (0.040)	6/39	21/39
	CR	1.38 (0.36)	0.875 (0.224)	1.59 (0.24)	25.9 (8.9)	35.1 (14.1)	2.4 (5.4)	1.192 (0.054)	0.149 (0.034)	2/24	12/24
	CR-AT	1.32 (0.28)	0.857 (0.219)	1.61 (0.26)	24.5 (3.5)	36.2 (10.3)	8.8 (3.0)	1.198 (0.131)	0.165 (0.046)	2/6	4/6

^1^The reported joint angles for walking exercise correspond to the moment of initial contact within the gait cycle and for the slow and fast popliteal angle tests correspond to the moment at which maximum HMTL is observed.

^2^This column indicates the number of limbs in which the maximum HMTL across all exercises (${\mathrm{HMTL}}_{\mathrm{max}}^{*}$) was provoked in each exercise (number of limbs/ total number of included limbs).

^3^This column indicate the number of limbs in which the observed HMTLmax exceeded the individual normative HMTLmax in each exercise (number of limbs/ total number of included limbs).

**Table 3:** Results of the healthy participants.

Exercise	Gait speed (1/s) (SD)	Step length (–) (SD)	Cadence (1/s) (SD)	Knee flexion^1^ (°) (SD)	Hip flexion^1^ (°) (SD)	Pelvis tilt^1^ (°) (SD)	HMTL_max_ (–) (SD)	HMTL_excr_ (–) (SD)	${\mathrm{HMTL}}_{\mathrm{max}}^{*}$ is provoked^2^
Slow Popliteal				14.9 (4.6)	70.1 (10.0)		1.328 (0.064)		1/10
Fast Popliteal				15.6 (5.1)	73.3 (11.0)		1.344 (0.076)		9/10
Walk 0.7 m/s	0.85 (0.05)	0.655 (0.060)	1.32 (0.13)	8.6 (2.6)	23.1 (3.8)	2.3 (2.2)	1.231 (0.051)	0.140 (0.009)	0/10
Walk 0.9 m/s	1.09 (0.06)	0.719 (0.041)	1.54 (0.11)	9.0 (2.7)	24.0 (4.1)	2.3 (2.2)	1.234 (0.052)	0.148 (0.008)	0/10
Walk 1.1 m/s	1.33 (0.10)	0.780 (0.044)	1.72 (0.10)	9.5 (2.8)	25.2 (4.2)	2.7 (2.4)	1.236 (0.054)	0.158 (0.009)	0/10
Walk 1.3 m/s	1.56 (0.12)	0.837 (0.056)	1.86 (0.11)	10.8 (3.1)	26.6 (4.5)	2.4 (2.2)	1.240 (0.051)	0.165 (0.010)	0/10
Walk 1.5 m/s	1.80 (0.10)	0.929 (0.056)	1.96 (0.10)	11.6 (2.9)	27.9 (5.9)	3.1 (3.3)	1.246 (0.053)	0.172 (0.013)	0/10
Walk fast	2.49 (0.25)	1.082 (0.129)	2.36 (0.15)	15.1 (3.6)	32.4 (5.9)	3.2 (2.6)	1.256 (0.052)	0.184 (0.017)	0/10
Walk with large steps	1.94 (0.42)	1.255 (0.094)	1.55 (0.27)	15.95 (5.8)	40.8 (9.03)	3.1 (4.0)	1.280 (0.060)	0.198 (0.020)	0/10

^1^The reported joint angles for walking exercise correspond to the moment of initial contact within the gait cycle and for the slow and fast popliteal angle tests correspond to the moment at which maximum HMTL is observed.

^2^This column indicates the number of limbs in which the maximum HMTL across all exercises (${\mathrm{HMTL}}_{\mathrm{max}}^{*}$) was provoked in each exercise (number of limbs/ total number of included limbs).

A significant main effect of participant type was observed (p = 0.002, α = 0.05). Across all exercises, the healthy group consistently displayed a larger average HMTL_max_ and a smaller standard deviation as compared to the patient group (Fig. 1). The significant interaction effect between group and exercise (p < 0.001, α = 0.05) showed that the difference in HMTL_max_ between the healthy and patient groups varied across exercise. The difference between HMTL_max_ of the healthy and patient group was not significant during walking at a comfortable speed (p = 0.041, α = 0.01) and walking fast (p = 0.014, α = 0.01), while it was significant during walking with large steps (p = 0.005, α = 0.01) as well as during slow and fast popliteal angle tests (p < 0.001, α = 0.01). Concerning the subgroups, the HMTL_max_ for the CR-AT group was consistently higher than that for the CR group across all exercises, with the exception of the slow popliteal angle test (Table 2).

Group comparisons of the maximum HMTL and reserve capacities showed that the average ${\mathrm{HMTL}}_{\mathrm{max}}^{*}$ and HMTL_rc_ was significantly smaller in patients (${\mathrm{HMTL}}_{\mathrm{max}}^{*}$: 1.231 [SD = 0.071], HMTL_rc_: 4.21% [SD = 2.47%]) as compared to healthy participants (${\mathrm{HMTL}}_{\mathrm{max}}^{*}$: 1.345 [SD = 0.078], HMTL_rc_: 7.71% [SD = 1.97%]) (p < 0.001, α = 0.05).

### HMTL; individual-level assessment

Tables 2 and 3 presents relevant HMTL_max_ and kinematic and spatiotemporal parameters for the patient and healthy participants, respectively. Among patients, the fast popliteal angle test provoked the largest HMTL across all exercises (${\mathrm{HMTL}}_{\mathrm{max}}^{*}$) in 20 out of 37 limbs. This was followed by the slow popliteal angle test, which provoked ${\mathrm{HMTL}}_{\mathrm{max}}^{*}$ in 12 out of 37 limbs, and walking with large steps did so in 6 out of 39 limbs. Fast walking provoked ${\mathrm{HMTL}}_{\mathrm{max}}^{*}$ in one out of 39 limbs, while walking at a comfortable speed did not provoke ${\mathrm{HMTL}}_{\mathrm{max}}^{*}$ in any limb (Table 2). In contrast, among healthy participants, the fast functional popliteal angle test provoked ${\mathrm{HMTL}}_{\mathrm{max}}^{*}$ in 9 out of 10 limbs, while the slow popliteal angle test did so in one limb (Table 3).

Out of 37 patient limbs, 27 and 28 limbs utilized an HMTL_max_ during the slow and fast popliteal angle test respectively which was larger than normative HMTL_max_ (what would have been required to adopt a gait pattern with normal, healthy knee and hip kinematics considering patient specific femur lengths) (Table 2). Walking with large steps provoked an HMTL_max_ larger than normative HMTL_max_ in 21 out of 39 limbs of the patients. Regarding the exercises of the current standard protocol—walking at a comfortable speed and walking fast—the numbers were lower, with 14 and 18 out of 39 limbs of the patients, respectively.

### Joint kinematics and spatiotemporal parameters

On average, patients walked slower, with smaller normalized step length, and with more knee and hip flexion at initial contact compared to healthy participants, under the same walking condition (Tables 1 and 2). Knee and hip flexion angles at initial contact were 22.2° [SD 8.3] and 31.7° [SD 7.6] in the patients when walking at a comfortable speed, and 10.8° [SD 3.1] and 26.6° [SD 4.5] in healthy participants while walking at 1.3 m/s.

During both the slow and fast popliteal angle tests, participants were instructed to flex their hips to approximately 90° and extend their knees as far as possible while maintaining hip flexion. Tables 2 and 3 show that healthy participants had smaller knee flexion angles (14.9° [SD 4.6]) for slow as compared to fast popliteal angle tests (15.6° [SD 5.1]) and larger hip flexion angles (70.1° [SD 10.0]) for slow as compared to fast popliteal angle tests (73.3° [SD 11.0]) when maximum HMTL was reached. Conversely, patients displayed knee flexion angles of 30.5° [SD 13.6] and 28.1° [SD 12.9] along with hip flexion angles of 57.7° [SD 11.2] and 57.8° [SD 12.0] during slow and fast popliteal angle tests, respectively.

## Discussion

In the current study, a new functional exercise protocol was introduced to improve the determination of HMTL_max_ in patients with a CNL and walking in crouch gait. Consistent with our initial hypothesis, the slow and fast functional popliteal angle tests and walking with large steps provoked significantly larger HMTL_max_ as compared to walking at a comfortable and fast speeds in all groups and sub-groups. Incorporating these exercises into the current standard 3D clinical gait analysis protocol reduced the number of patients classified as having short HMTL by 75%. Only one out of 22 patients was unable to perform the functional popliteal angle test, proving its feasibility in children and adults with a CNL and gait impairments.

**Figure 2:**
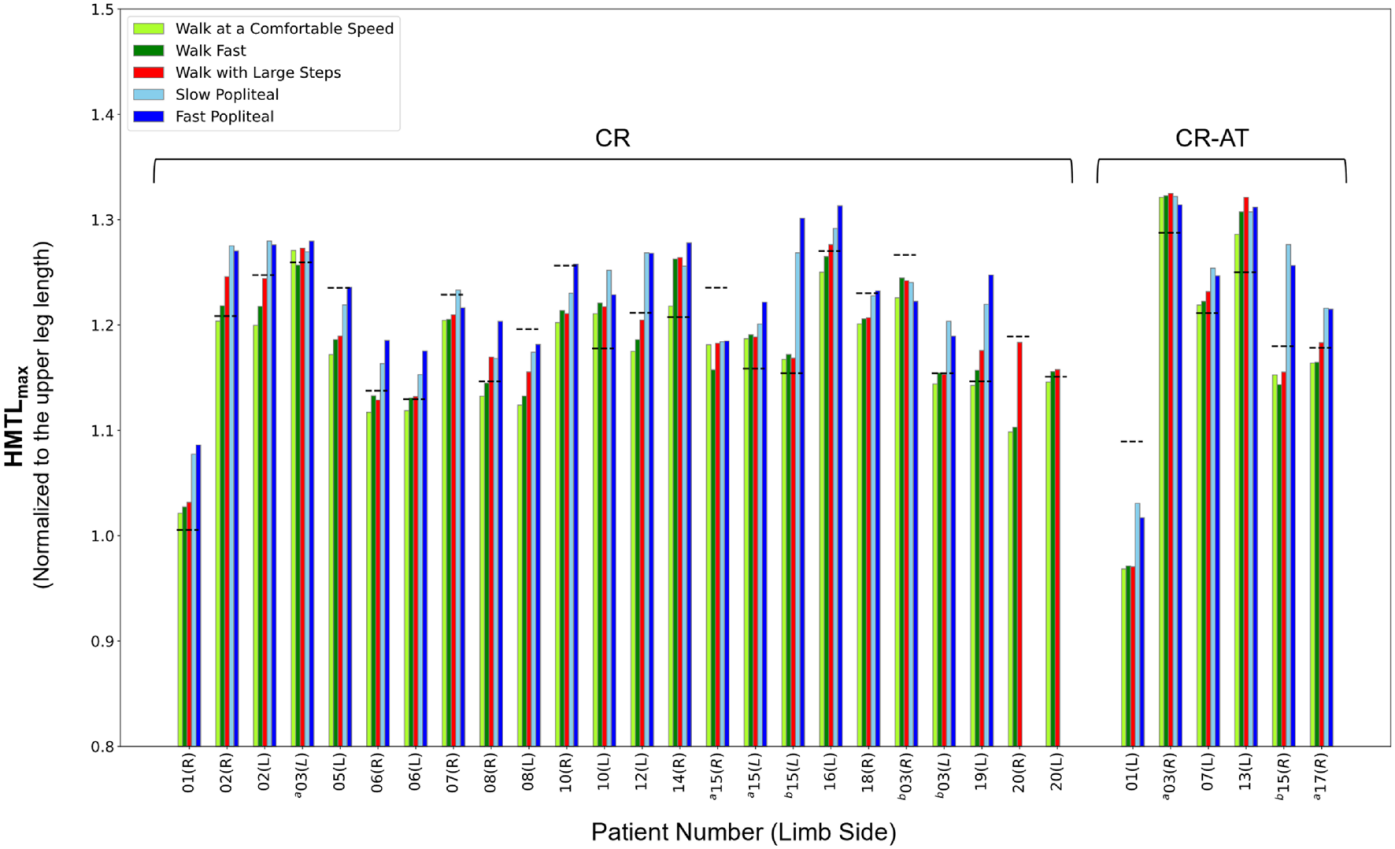
HMTL_max_ exhibited by individual limbs of patients with crouch gait (CR and CR-AT subgroups) across different exercises. The horizontal dashed lines represent the normative HMTL values for individual limbs. Patient number 27 was not able to perform the slow and fast popliteal angle tests. Patient identifiers are denoted with numbers, and 'a' or 'b' following the number indicates the same patient before and after the intervention, respectively.

Comparing HMTL_max_ across different exercises with the normative values of HMTL_max_ for individual patients (Fig. 2), reveals that adding the popliteal angle test and walking with large steps into the diagnostics of short HMTL, resulted in 4 out of 30 limbs being classified as having short HMTL. Conversely, including only the current standard protocol (walking at a comfortable speed and walking fast) resulted in 16 diagnoses of short HMTL. These results suggest that the adoption of the new protocol could potentially mitigate 12 false-positive diagnoses of short HMTL out of a total of 30 limbs. Our findings suggest that in previous studies which used the current standard protocol, patients received lengthening treatment while HMTL might not have been the main underlying cause of crouch gait. As a consequence, gait improvements have been reported to be rather small or lengthening treatment resulted in a decline of gait performance in about 15% of patients^13,15,17^.

**Figure 3:**
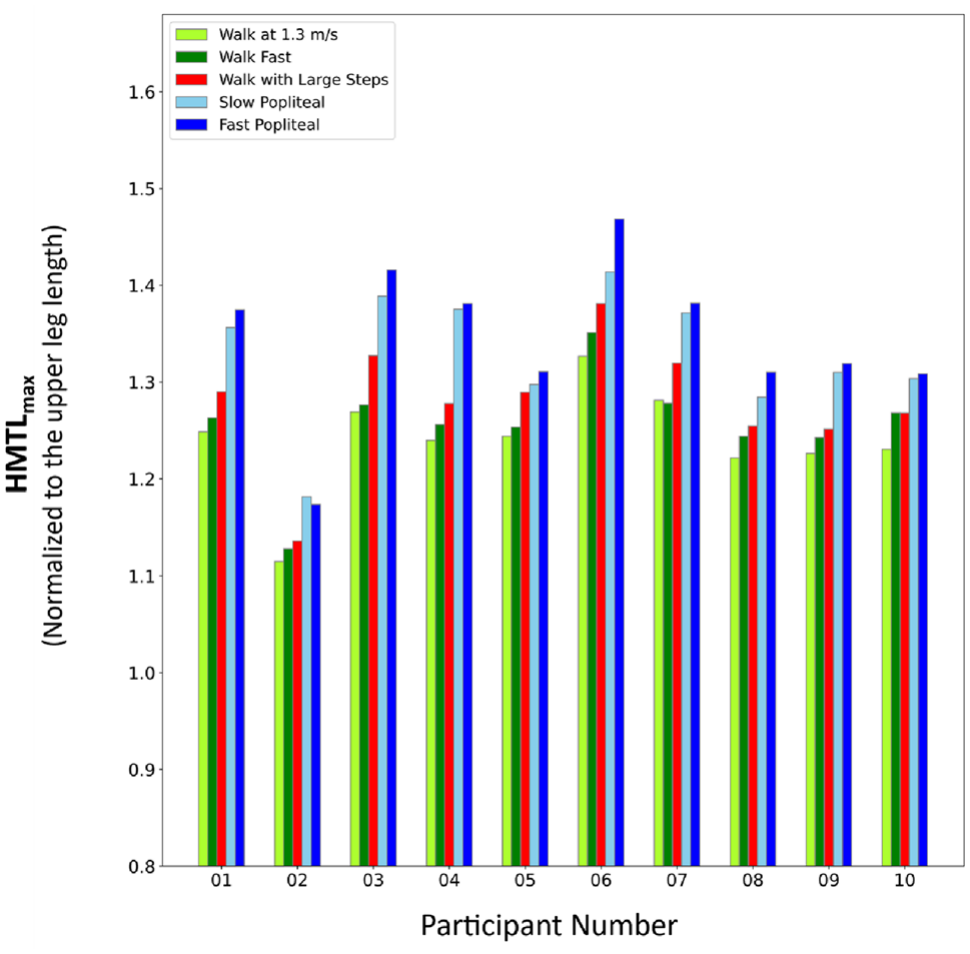
HMTL_max_ exhibited by individual limbs of healthy participants across different exercises. Patient identifiers are denoted with numbers.

Considerable inter-individual differences among patients in their responses to individual exercises were observed. While in all healthy participants, HMTL_max_ consistently increased across exercises and the fast popliteal angle test provoked ${\mathrm{HMTL}}_{\mathrm{max}}^{*}$ (except for one limb in which slow popliteal angle test provoked ${\mathrm{HMTL}}_{\mathrm{max}}^{*}$) (Fig. 3), there was no single exercise that consistently provoked ${\mathrm{HMTL}}_{\mathrm{max}}^{*}$ in each individual patient. This variability in patient responses suggests that individual specific neuromuscular impairments affect the patients’ capacity to reach ${\mathrm{HMTL}}_{\mathrm{max}}^{*}$ during different exercises. In addition, group level analysis showed that HMTL_max_ of healthy participants did not differ significantly with HMTL_max_ of patients when walking at a comfortable or fast speed. Our findings on individual specific responses to the functional exercise are in line with previously reported prevalence rates for short HMTL from studies using the standard protocol which ranged between 20 and 67% across studies^13,27,29,30^. We recommend therefore to incorporate all exercises of the presented protocol into diagnostics of individual patients with crouch gait to enhance diagnostic accuracy of short HMTL.

While most of the patients with crouch gait (CR and CR-AT subgroups), did not have absolute deficits in HMTL (their ${\mathrm{HMTL}}_{\mathrm{max}}^{*}$ were larger than their normative HMTLs), they did operate closer to their ${\mathrm{HMTL}}_{\mathrm{max}}^{*}$ when walking at a comfortable speed as compared to healthy participants (7.71% [SD 1.97%] vs 4.21% [SD 2.47%]). Operating closer to ${\mathrm{HMTL}}_{\mathrm{max}}^{*}$ (small HMTL_rc_) increases passive muscle–tendon forces, potentially slowing down or limiting the knee extension and hip flexion motion at the end of the swing phase. This observation is especially relevant because existing literature reports that patients with CP tend to have stiffer muscles compared to healthy controls^31^. Hence, even though some patients have sufficient maximum HMTL, large passive muscle–tendon forces might impair the hip flexion and knee extension motion in terminal swing when walking at comfortable speeds. These patients might benefit from treatments addressing muscle–tendon stiffness such as stretching exercises or eccentric hamstring exercises to facilitate longitudinal muscle growth^32^ and normal hip and knee joint kinematics.

The newly introduced HMTL_rc_ measure might also serve clinicians for early identification of patients at risk of developing crouch gait and in need for preventive interventions. Patients with a CNL often undergo repeated 3D clinical gait assessments. Incorporating the new exercise protocol into standard clinical care would allow to monitor HMTL_rc,_ identify patients with reducing reserve capacities at an early stage and provide preventive interventions. To establish the prognostic capacity of HMTL_rc_ in identifying the risk of developing crouch gait will be a main aim of future research activities. In addition, we will explore the capacity of the new exercise protocol and musculoskeletal modelling to better quantify the contribution of high passive hamstring muscle–tendon forces and hamstring muscle spasticity on crouch gait. The final goal is to develop more patient-specific treatments and improve treatment outcomes.

While this study enhances the diagnostics of short HMTL in patients with CNL, it does come with some limitations that should be considered when interpreting the results. Firstly, hamstring muscle spasticity might have affected HMTL_max_ estimates especially during the fast popliteal angle test and when walking at high speeds or with large steps. Our future research activities aim to establish whether the proposed exercise protocol combined with measures of hamstring muscle activity can improve diagnostics of hamstring muscle spasticity in patients with a CNL.

Another limitation is the lack of age-matching between the patients and healthy participants. Although, we normalized HMTL parameters as well as spatiotemporal parameters to the femur length to elicit the effect of the differences in height, other parameters might be different among individuals with different ages and affect the outcome measures and interpretation of the results. In addition, in this study we used a generic musculoskeletal model which does not account for the bone abnormalities that are common in patients with CP^6,33^. In addition, three patients were included in this study twice (before and after an intervention), resulting in six datasets. This could potentially affect the accuracy of the results, as these paired datasets may be interrelated in ways not accounted for in the analysis.

In conclusion, this study introduced an innovative functional exercise protocol that enhances the diagnostic accuracy of short HMTL in patients with CNL. Incorporating slow and fast functional popliteal angle tests, as well as walking with large steps into 3D clinical gait analysis improves identification of HMTL_max_ and significantly reduces the number of false-positive diagnoses of short HMTL. The new functional exercises also account for patient-specific variability in neuromechanical impairments, thereby offering a more targeted approach for diagnostics and subsequent treatment planning. As part of our upcoming efforts, we will work on extending the exercise protocol to study its accuracy for hamstring spasticity assessment and to identify its contribution in developing crouch gait.

### Supplementary Information

Supplementary Information 1.

Supplementary Information 2.

## Data Availability

The data that support the findings of this study are available from the corresponding author on request.
