# Supplementary material for: Functional popliteal angle tests improve identification of short hamstring muscle–tendon length in patients with a central neurological lesion
Source: Sci Rep. 2023 Nov 22;13:20510. doi: 10.1038/s41598-023-47667-8 (PMC10665385; doi:10.1038/s41598-023-47667-8)
Supplement: Supplementary file 2 — Supplementary Information 2. [file 41598_2023_47667_MOESM2_ESM.docx]

***Description of the entire functional exercise protocol***

The current study is part of a larger project and only those details relevant to this paper are described in the main manuscript. The entire experimental protocol includes these steps:

**Functional Exercises** (Supplementary Figure 1)

1. Heel rise test: Complete 5 repeats.
2. Squat test
3. Functional popliteal angle test: 1. Slow: Patient stands on one leg, supported by a caregiver. Patient flexes the hip to 90 degrees and extend the knee 3-5 times slowly to the maximum extent. 2. Fast: Patient stands on one leg, supported by a caregiver. Patient flexes the hip to 90 degrees and extends the knee 3-5 times as fast as possible to the maximum extent
4. Functional Duncan-Ely test: 1. Slow: Patient stands on one leg, supported by a caregiver. Extend the hip as far as possible, then flexes the knee 3-5 times slowly to the maximum extent. 2. Fast: Patient stands on one leg, supported by a caregiver. Extend the hip as far as possible, then flexes the knee 3-5 times as fast as possible to the maximum extent
5. Gastrocnemius stretch test: advance the non-affected leg forward, extending the affected leg as far rearward as possible while maintaining heel-to-ground contact.
6. Walk at a comfortable speed: Complete 15-20 steps.
7. Walk as fast as possible: Complete 15-20 steps.
8. Walk with large steps: Complete 15-20 steps.


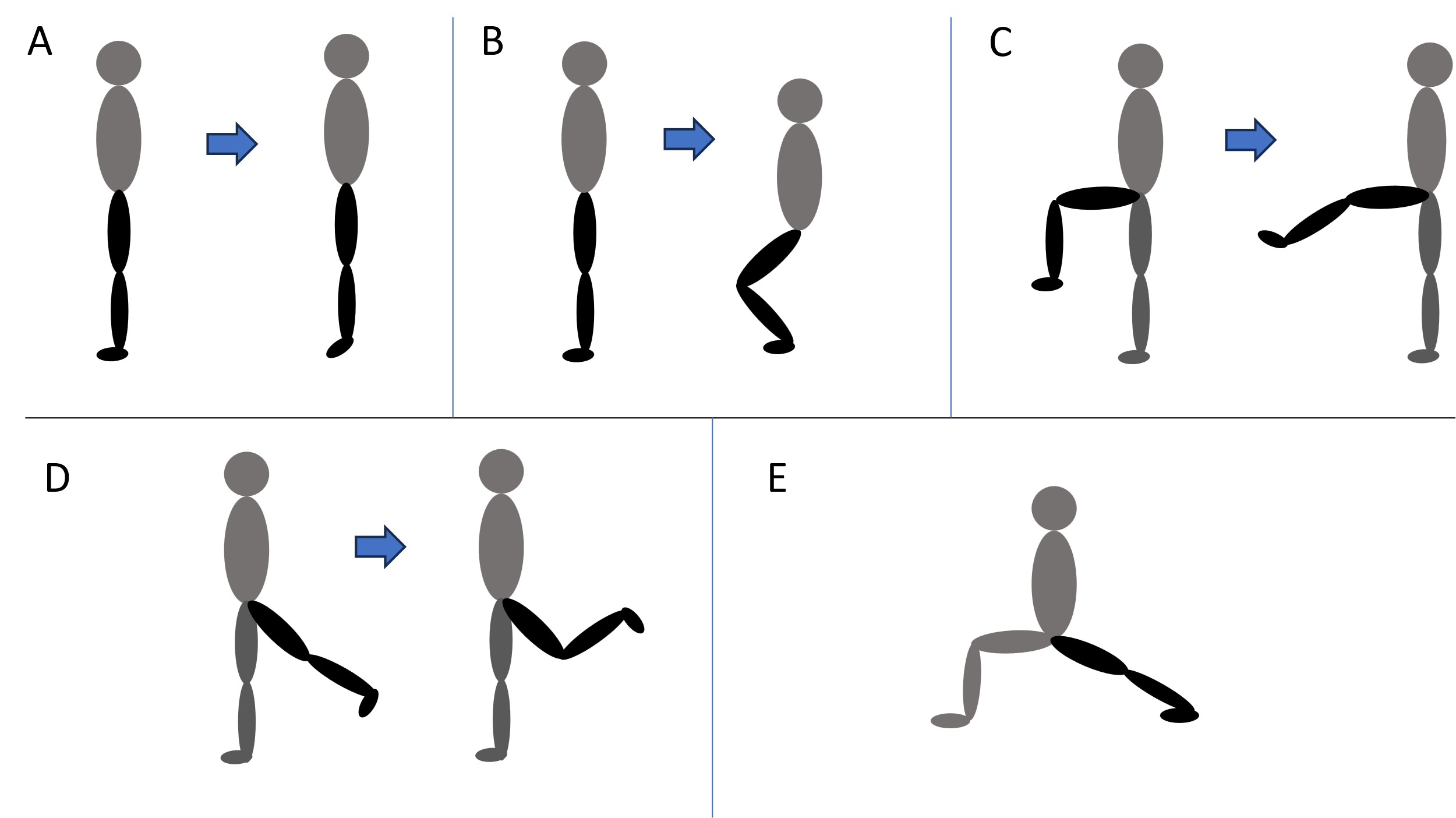


Supplementary Figure 1. Illustration of the Functional Exercises Included in the New Functional Exercise Protocol. A: Heel Rise, B:Squat, C: Functional Popliteal Angle Test, D: Functional Duncan-Ely test, E: Gastrocnemius Stretch Test.

**Physical Assessment:** (Conducted by a Movement Scientist)

1. Passive Duncan-Ely^1^: Position the participant in a prone posture. Flex the knee passively both slowly and quickly to the furthest extent without moving the hips.
2. Passive Popliteal Angle^1^: Position the participant in a supine position. Flex both the hip and knee to 90° while keeping the contralateral leg extended. Extend the flexed knee slowly and quickly to the furthest extent possible..

**Data Acquisition:**

During all functional exercises, acquire 3D marker position data. Use 18 reflective markers, including 16 markers from the Plug-in-Gait Lower Body model and 2 extra markers on the medial knee. For static calibration, employ medial knee and femur markers to calibrate the knee flexion-extension axis. Remove 2 extra markers following statistical calibration.

Acquire EMG data for gastrocnemius medialis, soleus, tibialis anterior, vastus medialis, rectus femoris, and semitendinosus at a sampling rate of 2000Hz. Place sEMG electrodes according to SENIAM guidelines^2^.

***Inter-individual comparison between the length of medial and lateral hamstring muscles***

Supplementary Figure 2 represents the variations in muscle length for the semimembranosus, semitendinosus, and biceps femoris (long head) across five exercises in the newly developed functional test protocol for healthy individuals and patients. Across all exercises, each of these three muscles demonstrated comparable trends in length changes. The maximum elongation in muscle length was observed during the fast popliteal angle test, followed by slow popliteal angle test, walking with large steps, walking fast, and finally walking at a comfortable speed for patients or at a speed of 1.3 m/s for healthy participants. Our analysis specifically targeted biarticular hamstring muscles that affect both the knee and hip joints; hence, the short head of the biceps femoris, which exclusively affects the knee joint, was excluded from the analysis.


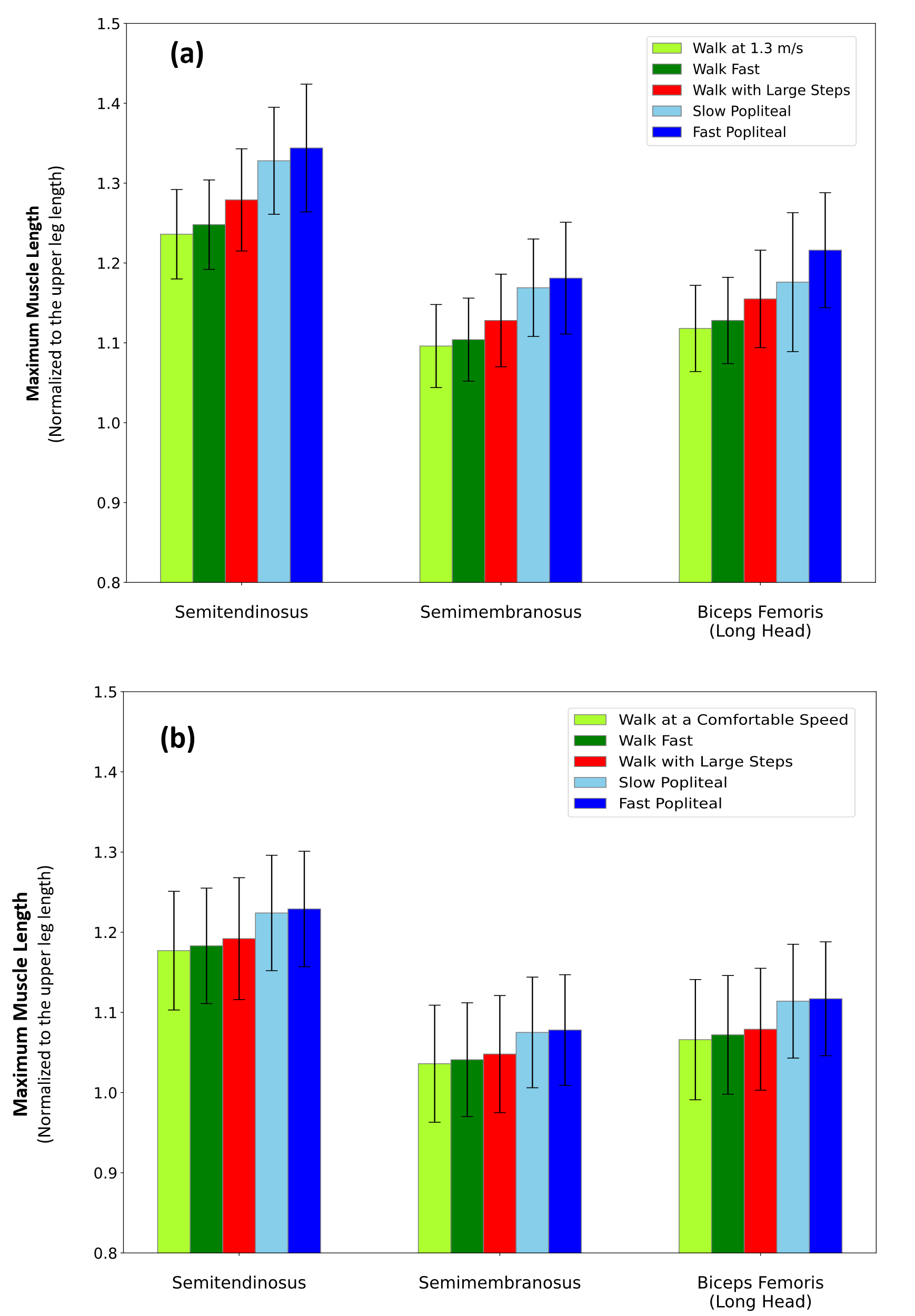


Supplementary Figure 2. Mean and Standard Deviation of Maximum Muscle Lengths Provoked Across Various Exercises of the New Functional Test Protocol. Muscles analyzed include the semitendinosus, semimembranosus, and long head biceps femoris for (a) healthy and (b) patient groups.

***Normative HMTL_max_ generated for healthy participant***

Healthy lower limb kinematics were imposed on healthy participants’ scaled OpenSim models to validate our approach of generating patient-specific normative HMTL_max_ values. The generated normative HMTLs were compared with the observed HMTL_max_ values at a walking speed of 1.3 m/s. Supplementary Table 1 presents both the generated normative HMTL_max_ and the observed HMTL_max_. The mean difference between the normative HMTL_max_ and the HMTL_max_ when walking at 1.3 m/s was 1.96 % [SD=1.40 %] of the normalized HMTL_max_. These findings validate the normative HMTL_max_ as a reliable metric for establishing patient-specific HMTL threshold values required to achieve knee and hip joint kinematics at initial contact comparable to a healthy walking pattern.

Supplementary Table 1: Normative HMTL_max_ values and observed HMTL_max_ during walking at 1.3 m/s, along with the difference between these values, for individual healthy participants.

| Participant’s Number | HMTL_max_ during walking at 1.3 m/s [-] | Normative HMTL_max_ [-] | Difference [%] |
| --- | --- | --- | --- |
| 01 | 1.248 | 1.274 | 2.04 |
| 02 | 1.115 | 1.143 | 2.50 |
| 03 | 1.269 | 1.248 | 1.62 |
| 04 | 1.240 | 1.232 | 0.64 |
| 05 | 1.244 | 1.271 | 2.20 |
| 06 | 1.327 | 1.253 | 5.58 |
| 07 | 1.281 | 1.299 | 1.33 |
| 08 | 1.221 | 1.243 | 1.72 |
| 09 | 1.226 | 1.216 | 0.87 |
| 10 | 1.230 | 1.244 | 1.07 |
| Mean (SD) | 1.240 (0.051) | 1.242 (0.042) | 1.96 (1.40) |

***Joint kinematic of the representative healthy participant used to generate normative HMTL***

Healthy lower limb kinematics from a single gait cycle of a representative healthy participant is shown in Supplementary Figure 3.


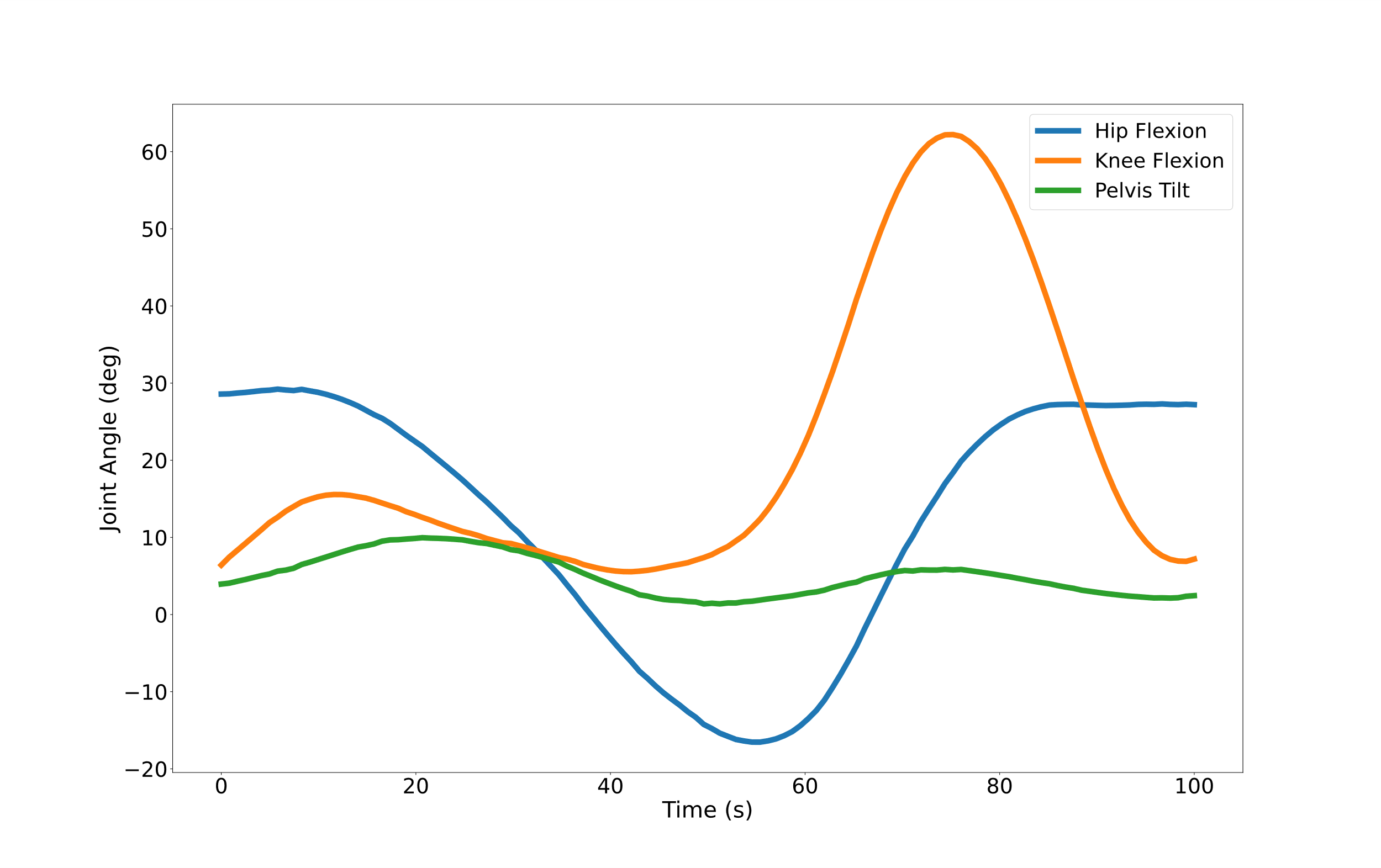


Supplementary Figure 3. Hip, knee, and pelvis angles of single gait cycle of a healthy participant.
